# Nurses’ perception of work and life under COVID-19 pandemic conditions: a qualitative study

**DOI:** 10.3389/fpubh.2023.1292664

**Published:** 2023-12-15

**Authors:** Esmaiel Maghsoodi, Zohreh Vanaki, Eesa Mohammadi

**Affiliations:** Department of Nursing, Faculty of Medical Sciences, Tarbiat Modares University, Tehran, Iran

**Keywords:** COVID-19, nursing, perception, work, life, qualitative study

## Abstract

**Objectives:**

Although nurses work and live in special and stressful conditions due to the nature of their profession, in periods of crises and pandemics, when the work pressure on nurses and the public’s need for professional services increases to extraordinary and incomparable levels compared to customary conditions, their work and life situation becomes entirely different. Therefore, what nurses experienced in the COVID-19 pandemic went beyond the typical challenges of their work environment. This study was conducted to discover nurses’ perceptions of work and life during the COVID-19 pandemic.

**Methods:**

This qualitative study was conducted with a conventional content analysis approach on 16 nurses working in the inpatient wards during the COVID-19 pandemic in Iran. Data were collected through unstructured, individual, and in-depth interviews between August 2020 and June 2023 and were analyzed using content analysis with the conventional (inductive) approach of Granheim and Lundman.

**Results:**

Data analysis led to the extraction of 11 subcategories, namely, “feeling a lack of support and understanding from managers,” “team cooperation and communication challenges in difficult work conditions,” “shadow of burnout,” “shortage inequity,” “dissatisfaction with unfair wages and benefits,” “not having their work and sacrifices appreciated,” “suffering and fatigue of using personal protective equipment (PPE),” “deprivation of entertainment and rest,” “fear of illness and imminent death,” “low resilience,” and “deteriorating life conditions” These 11 subcategories led to the following themes: “unsafe work environment” and “the shadow of suffering and death.”

**Conclusion:**

Nurses working in COVID-19 wards in Iran worked in an unsafe work environment during the COVID-19 pandemic and had to deal with suffering and fear of death. It is necessary to pay attention to the needs and problems of nurses, and healthcare organizations must provide the required support to maintain the physical and mental health of nurses during epidemics.

## 1 Introduction

The spread of COVID-19 was so fast and surprising that the World Health Organization declared it a global pandemic crisis (1). In addition to infecting a large number of people and causing physical and psychological effects in society, nurses, as health advocates on the front line of fighting the disease, suffered many adverse effects of the disease (2). Studies show that the mental side effects of COVID-19 in nurses are far more permanent than the physical side effects (3). Prevalence of psychological and emotional problems, such as stress, anxiety, depression, fear of illness or death of loved ones, as well as professional issues such as job burnout, fear of going to work, symptoms of post-traumatic stress syndrome, and other problems caused by the challenges of working in COVID-19 wards were prominent (3, 4). Arnetz et al. (5), in their study of participants from the Michigan Chapter of the American Nurses Association (ANA), the Michigan Organization of Nursing Leaders (MONL), and the Coalition of Michigan Nursing Organizations (COMON), have reported that most nurses became afraid to go to work, that they were also relatively less worried about the lack of use of personal protective equipment, and that most nurses were concerned about their family and close friends becoming sick (6). The problems that the vast majority of healthcare systems around the world were dealing with at the beginning of the outbreak and the rapid spread of COVID-19 included a lack of patient care equipment and personal protective equipment for the staff, lack of hospital space, high workload, and limited knowledge and information about the cause of the disease, its pathophysiology and transmission routes, which caused physical and mental problems that have since become emotional and social issues for nurses (7). In most disasters and infectious diseases that have emerged as epidemics, nurses have acted selflessly to provide services, considering their moral and professional responsibility, and even sometimes neglected themselves and their needs (6). Undoubtedly, if this sense of responsibility does not exist in nurses, it would not be possible to continue providing care in the stressful conditions of a disease such as COVID-19 and the hard-working conditions caused by it (8). In different cultural and social contexts, nurses have other working conditions and characteristics (9). As a matter to consider, during the COVID-19 pandemic, the stigma of being a carrier nurse has been reported in countries such as Iran, Indonesia, and Italy (10–13). The quality of nurses’ performance and physical and mental health are related to proper working conditions. However, studies have shown that the COVID-19 pandemic is associated with the working conditions and organizational problems caused by it and the development of psychological issues in nurses (14).

One of the issues that exists as a basis for the difficulty of working conditions is the need for similar management experience for managers during the COVID-19 pandemic. In the context of the experiences of nursing managers during the COVID-19 pandemic, a study was conducted by White et al. (7) that refers to the managers’ focus on psychosocial support for nurses while they were exhausted and stressed. Considering that these experiences take place in the form of behavioral actions between managers and nurses, the experiences of behavioral interactions in this regard can be different in different contexts, as in the study of Moradi et al. (15), which reported an ineffectiveness of health organizations in supporting nurses. This ineffectiveness of organizations is more or less related to the need for more support from managers (15). Studies on nurses’ working and living conditions have been conducted separately and on limited variables, and sometimes studies have been conducted on a specific issue, such as the psychological challenges faced by nurses or the stigma of treating a certain disease (11, 14, 16). However, the neglected link in this field is the awareness of the lived experiences of nurses in a broader way regarding their living and working conditions during the COVID-19 pandemic, considering that these issues are different in different cultures and contexts. Therefore, it is necessary to carry out qualitative research to reveal these experiences in the field and culture of Iran. Therefore, this study was conducted to discover nurses’ perceptions of work and life during the COVID-19 pandemic in Iran.

## 2 Materials and methods

### 2.1 Design

The present study is a qualitative study that was conducted with the conventional content analysis approach. Content analysis is a qualitative descriptive research method that was suitable to identify nurses’ perceptions of work and life during the COVID-19 pandemic (17).

### 2.2 Participants and setting

The participants of this study were 16 nurses working in COVID-19 inpatient wards. Of the total, seven nurses were men and nine were women. The participants were employed in educational and private hospitals in Tehran, and East and West Azerbaijan provinces and were selected by purposive sampling. The criteria for entering the study included at least 1 month of work experience in COVID-19 inpatient wards, the ability to communicate, and the willingness to share their experiences. The characteristics of the participants show the diversity in the selection of nurses (Table 1). The participants in this study were 16 nurses working in COVID-19 wards. They had an average age of 36.47 ± 7.88 years and an age range of 26 to 51 years. There were seven men and nine women. Of the total, 13 participants had a bachelor’s degree and 3 had a master’s degree in nursing. A total of 11 participants were married, and 5 were single. Seven worked as nurses at ICU COVID-19 wards, seven participants were nurses of COVID-19 wards, and two were nursing managers (Table 1).

**Table 1:** Demographic characteristics of the participants.

Variable		Number
Age	24–30	7
	30–40	6
	>40	3
Covid-19 work experience (months)	1–6	2
	6–18	4
	>18	10
Nursing Experience (years)	<5	4
	5–15	6
	>15	6
Sex	Male	7
	Female	9
Education	Bsc	13
	Msc	3
Marital status	Married	11
	Single	5

### 2.3 Data collection

Data were collected through unstructured, individual, and in-depth interviews between August 2020 and June 2023. After obtaining the necessary permits from the university and the research areas, the researchers went to the research areas. They inspected and visited them closely following health protocols and invited potential participants to participate in the study and conduct interviews. The participants determined the interview conditions, including the time, place, and type of interview (in person or virtually). Due to the COVID-19 pandemic and the need to comply with health principles, seven interviews were conducted through Skype and phone calls, and nine interviews were conducted in person by observing the use of personal protective equipment. The flexibility of unstructured interviews helped explore individual issues raised during the discussions and deeply reflected the participants’ experiences of the study phenomenon. To gain the trust of the interviewees, the interviewer first introduced him/herself and then explained the method of conducting the interview and the objectives of the study. The interviewer obtained informed consent from the interviewees and their demographic characteristics such as age, marital status, educational qualification, work experience, and work experience in the hospital department dedicated to COVID-19. The mean duration of the interviews was 64 min, with the minimum interview time at 50 min and the maximum time at 80 min.

The interviews began with an open-ended question: “Describe a day at work during the COVID-19 pandemic?” Then, questions gradually focused on nurses’ working and living conditions. According to the participants’ answers, follow-up, probing, and exploratory questions were asked such as: “Can you explain more?” “What do you mean?,” and “Can you tell me a practical example from this experience so that I can better understand what you mean?.” After obtaining permission from the participant, the interviews were recorded by a recording device. Then, immediately after the interview, they were transcribed word for word. Data saturation was achieved in the 14th interview, and two more interviews were conducted to improve the comprehensiveness of data collection.

### 2.4 Data analysis

In this research, the qualitative research method of content analysis with the conventional (inductive) approach of Granheim and Lundman was used to analyze the data at different levels. This method includes selecting semantic units, condensing semantic units and coding, and creating subcategories and categories (themes).

**Figure 1:**
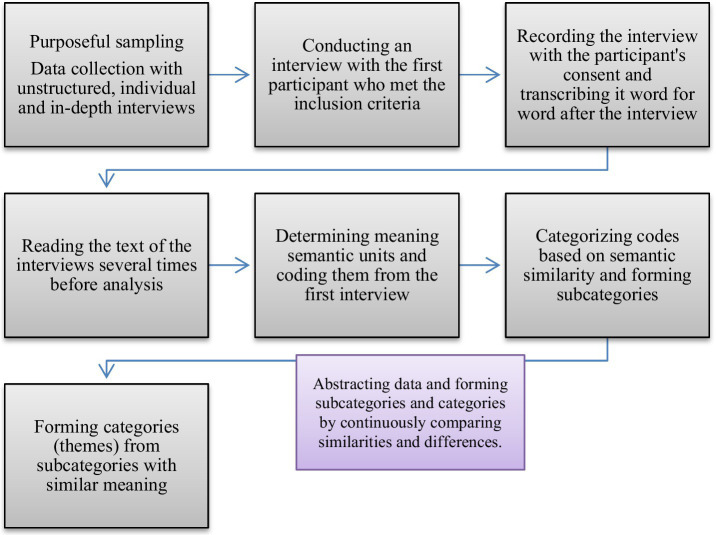
Flowchart of method stages.

After each interview, the researcher transcribed the audio file containing information word for word and converted it into text format. In this method, the entire text of each interview is called the unit of analysis, which must be coded and classified during content analysis. For this purpose, the whole text was read several times before starting the analysis, and the coded units were selected as the central part of the content analysis. The entire interview text was considered a unit of study, and then the analysis process continued with the classification of codes (Table 2). During the formation of subcategories and categories, continuous comparison was made to check the ability to differentiate between them. Subcategories and categories were developed by continuously comparing similarities and differences, and data abstraction was performed [(18); Figure 1]. All interviews were conducted and analyzed under the supervision of a research team with sufficient experience in both qualitative research and nursing.

**Table 2:** The examples of coding.

Quotations	Original initial codes
They did not distribute the benefits and wages of Corona fairly. I was a complainer and even brought it up, but unfortunately, we are in an administrative system where we follow up on financial issues a lot, probably the managers will bring up a series of other issues and the subsequent troubles you say, do not worry about it. (P6) [sic]	Fear of accountability by managers
At the beginning of the crisis, there was a shortage of personal protective equipment, and then there was no attempt by the managers to provide standardized personal protective equipment…masks were unfortunately not the standard of Covid-19 ward. (P7) [sic]	Failure to provide standard personal protective equipment
At the beginning of Corona, I said that I want to go to the emergency room from this department, I have been working here for 7 years. Matron said that you are running away, I told him that I am no longer useful here. Who is listening, as if not as if (P10) [sic]	Managers’ lack of attention to the demands of nurses
If we got sick and applied for sick leave, the officials, especially the matron, would say that you are making yourself sick, go on sick leave. They no longer give sick leave to the result of PCR, and their trusted physician had to confirm that we were infected. (P10) [sic]	Accusation of harassment from managers when nurses requested sick leave
It happened a lot that they did their own work and then went with the help of a colleague who was busier and did not put extra pressure on us. Sometimes there are colleagues who grumble, well, difficult situations and people are different, their behavior is also different in difficulty. (P3) [sic]	Relative cooperation and coordination between colleagues in difficult working conditions
There is cooperation and non-cooperation. If they do not help me; I will not help them. This kind of unprofessional behavior in the work environment makes person very nervous; For example, we draw lots to divide the work of patients, colleagues cheat so that they do not take sick patients, such small things make people nervous. (P8) [sic]	Fraud and unusual behavior of colleagues in determining work shifts
Despite the hard-working conditions we had during the COVID-19 era, I was drained physically due to the high workload, and from a mental point of view, because young patients were dying or having problems at work and on the sidelines, it made me more tired.(P3) [sic]	Excessive physical and mental fatigue at work
I’m tired of everything. I want to resign, but I’ve worked in this system for ten years. Hope for the future sometimes comes to me. (P10) [sic]	Willingness to leave the job
Despite the bad working conditions, the degradation of social status during the Covid-19 era, low salaries and benefits, and humiliation by the officials, I became completely unmotivated in my work. (P10) [sic]	Lack of motivation
The shortage of personnel was felt in COVID-19 couriers, and that was due to the illness of some colleagues and the lack of fresh personnel.(P9) [sic]	Lack of workforce
Most cases of co-workers happened in the rest room. All those who used the X-room, all of them tested positive in one week. Our next ward is the post CCU, the ward that is mostly non-covid, our rest room in the ICU is shared with them, which is separated by two closets as long as one meter, which is exchanging air from above. (P6) [sic]	Non-standard space of wards and personnel restrooms
Unfortunately, there was no proper ventilation in the department where we worked, and there was always a virus in the ward’s air, which created bad conditions for us and the patients. (P7) [sic]	Improper ventilation of the ward, leading to the spread of the disease
The initial crisis was such that masks, protective clothing, and even gloves could not be found all of a sudden, and our hospital also faced a shortage, and we were forced to use quotas. (P7) [sic]	Lack of personal protective equipment and medical equipment
Unfortunately, our salaries increased a little, but not that much; for example, With the hard working conditions we had and the deal was for our lives, we should have a much higher salary. (P8) [sic]	Imbalance between nurses’ working conditions and salaries and benefits
For the small salary that we were entitled to, the managers used to make us pay it on time. Regarding fee for service, it was updated, but it was a third of a year ago, they said that the hospital’s income has decreased. Salaries and benefits had actually decreased, but the cost of living had increased. (P10) [sic]	Low salaries and benefits and resulting mental and physical problems
The situation was absolutely critical and we thought that we were in a war situation. I felt that now I am not speaking for myself, my colleagues were sacrificing more than they could (P7) [sic]	Imagine and describe the scene of the sacrifice of nurses on the battlefield.
This situation is like wartime, with this difference, those who went to the front and returned, everyone went to meet them and make them happy. But we are very strange, we do not need to be taken over, at least they do not destroy and reject us. (P5) [sic]	The popularity of soldiers versus the rejection of nurses
We had to use a protective cover, special warm clothes, some of which were non-standard and wearing them was painful. (P7) [sic]	Wearing non-standard clothes with disconmfort
Exactly what the pictures and videos showed, the traces of the masks on the face and the wounds were created, it took half a day and a day to return to normal. Also, some gloves and clothes caused eczema and sensitivity on our skin. (P6) [sic]	Wounds and traces of masks causing skin sensitivity from non-standard clothes and masks and too much washing.
Physically, I said that we are worn out due to excessive fatigue and work pressure, I have back pain, I have varicose veins. (P10) [sic]	Low back pain and varicose veins caused by hard work.
And after the spread of covid-19, I get headaches that last for a certain number of hours or with a wave, because when we give old oxygen to the lungs, headaches occur, I have that hypoxia. (P6) [sic]	Headache caused by prolonged use of masks.
After the outbreak of covid-19, going to the market and shopping and such things, such places, are almost canceled for us, unless we have to go out. The fun we had before, the park or a place to go for a walk, these are mostly canceled. (P1) [sic]	There is a lack of entertainment and fun in life.
We nurses are currently unable to travel, both because of the spread of the disease and because of the many shifts. The working conditions have become so difficult due to the illness of colleagues and the lack of manpower that we cannot think about fun and travel. (P4) [sic]	Lack of travel and entertainment due to the conditions of the coronavirus disease and multiple shifts.
I’ve been under a lot of mental stress since the severe lung complications caused by COVID-19; it always made me have nightmares that God forbid I get infected again, and this time I do not survive and die. In other words, I always feel doomed, and I think I will probably get stuck and die these days. (P8) [sic]	Fear of catching a disease and fear of imminent death due to infection.
I am afraid of my family, friends and people who are dear to me getting sick. Now the fear is more because my job is related to health and care, but I cannot do anything in case of severe conflict with my loved ones. And it is not in my hands whether they get sick or recover, and God knows how stressful this situation is for me. (P15) [sic]	Fear of infecting family and friends.
Once, my father got sick and his lungs were nearly 70% affected and he could not breathe well. My heart was empty and I said that he will die, I was very scared, I said that I wish I was in my father’s place. The stress was really deadly, I died and revived a thousand times in those few days. (P13) [sic]	Fear of imminent death of oneself and loved ones.
I have the experience of heavy shifts. The patients were in a very bad condition, most of them were in a coma phase. Unfortunately, our shifts were back to back, well, I was always on shift, and I was dealing with a sick patient, and this caused a hidden stress to come to me. (P7) [sic]	High workload and stress.
I was very sad when a young man suffered in front of my eyes, who was not like a cancer patient whose fate is likely to be known, this young man dies. A few days ago, two young patients died, I was very sad, I went home, I had become aggressive. These conditions affect our psyche, either we become aggressive or depressed. (P5) [sic]	Aggression caused by lower tolerance thresholds.
I could not bear all these troubles in crowded couriers like the fifth courier. My sister’s husband was young, God’s servant died. At work, at home, instead of being a pillar for those around me, I was all complaining, why did this disease come. What sin did we commit?(P15) [sic]	Reduced tolerance and increased whining.
Well, all this stress and fear of getting infected disrupts life, I do not enjoy anything. I do not enjoy my work at work, I do not enjoy anything in my daily life, for me, life has become like hell. (P4) [sic]	Extinction of pleasures and “poisoning of life.”
Living conditions had become extremely difficult, you experienced it yourself, and we could not even sit with our families without masks. The whole life plans for everyone and for me are messed up. (P11) [sic]	Difficult living conditions after the pandemic.

### 2.5 Rigor

To verify the accuracy of the study results, four Guba and Lincoln criteria, namely, “validity,” “adaptability,” “reliability,” and “transferability” were evaluated (19). To ensure the credibility of the findings, it is possible to mention the researcher’s long-term interaction with the data and the correctness of coding and conceptualization was checked with the participants and by a third party. After extracting the primary codes, the coded texts were given to some nurses participating in the study to check the accuracy of the interpretations. A peer check was used to ensure confirmability. In this way, four foreign peers experienced in conducting qualitative research evaluated some of the interviews and the coding, classification, and data abstraction process. They confirmed the accuracy of the data analysis. The study’s dependability was also ensured through data analysis by the authors sharing their results. The nurses who checked the accuracy of the interpretations confirmed the consistency of the classes, and the researchers agreed with this during a meeting that was held on data analysis. Sampling with maximum diversity in terms of sex, education level, and work experience and comparing the study’s findings with the results of other studies were also done to ensure transferability.

## 3 Results

This study was conducted to answer the question of what were nurses’ perceptions of work and life under COVID-19 pandemic conditions. After analyzing 16 interviews, 200 initial codes were extracted. After reviewing and rechecking the codes in terms of duplication and merging some of the codes, the number decreased to 71 principal codes. In the next step, data analysis led to the extraction of 11 subcategories and two main themes. The first theme, titled “unsafe work environment,” had features in the form of seven subcategories that were understood and experienced by nurses: “feeling of lack of support and understanding from managers,” “team cooperation and communication challenges in difficult work conditions,” “shadow of burnout,” “shortage inequity,” “dissatisfaction with unfair wages and benefits,” “not seeing and appreciating nurses’ war sacrifice,” and “suffering and fatigue of using personal protective equipment (PPE).” The second theme, titled “the shadow of suffering and death on life,” had features in the form of four subcategories that were understood and experienced by nurses: “deprivation of fun and rest in life,” “fear of illness and imminent death,” “low resilience,” and “deteriorating conditions of life” (Table 3). These concepts indicate that nurses struggled to endure the harsh working and living conditions during the COVID-19 pandemic, mainly perceived as an unsafe work environment and difficult life combined with the fear of death. In the following sections, the themes are explained and described with sub-themes and related quotes.

**Table 3:** Conceptualization process resulting from data analysis in this study.

Original codes	Subcategories	Themes
Fear of accountability by managers	The feeling of lack of support and understanding from managers	Unsafe work environment
Failure to provide standard personal protective equipment		
Managers’ lack of attention to the demands of nurses		
Accusation of harassment from managers when nurses requested sick leave		

Relative cooperation and coordination between colleagues in difficult working conditions	Team cooperation and communication challenges in difficult working conditions	
Fraud and unusual behavior of colleagues in determining work shifts		

lack of motivation	The shadow of burnout	
Excessive physical and mental fatigue at work		
Willingness to leave the job		

Lack of workforce	Shortage inequity	
Non-standard space of wards and personnel restrooms		
Improper ventilation of the ward that causes the spread of the disease		
Lack of personal protective equipment and medical equipment		

Imbalance between nurses’ working conditions and salaries and benefits	Dissatisfaction with unfair wages and benefits	
Low salaries and benefits and resulting mental and physical problems		

The popularity of soldiers versus the rejection of nurses	Not having their sacrifices appreciated	
Imagine and describe the scene of the sacrifice of nurses on the battlefield.		

Wearing non-standard clothes with torment	Suffering and fatigue in using personal protective equipment (PPE)	
Wounds and traces of masks causing skin sensitivity from non-standard clothes and masks and washing too much.		
Low back pain and varicose veins caused by hard work		
Headache caused by prolonged use of the mask		
Lack of entertainment and fun in life	Deprivation of entertainment and periods of rest	The shadow of suffering and death
Lack of travel and entertainment due to the conditions of the coronavirus disease and multiple shifts		

Fear of catching a disease and imminent death due to infection	Fear of illness and impending death	
Fear of infecting family and friends		
Fear of imminent death of oneself and loved ones		

High workload and stress	Low resilience	
Aggression caused by lowering the tolerance threshold		
Reduce tolerance and whining.		

Extinction of pleasures and poisoning of life	Deteriorating life conditions	
Difficult living conditions after the pandemic		

### 3.1 Unsafe work environment

Nurses understood and expressed the idea of an unsafe work environment as follows: a feeling of lack of support and understanding from managers, team cooperation, communication challenges in demanding work conditions, the shadow of burnout, shortage inequity, dissatisfaction with unfair wages and benefits, not having their sacrifices appreciated, and suffering and fatigue of using personal protective equipment (PPE).

#### 3.1.1 Feeling of lack of support and understanding from managers

In addition to the high workload and social challenges that existed for nurses during the outbreak of the COVID-19 disease, nurses claimed to feel that they were not supported and understood by managers, which was expressed in the form of managers’ lack of empathy with them. They said, for example, that nurses faced accusations of pretending to be sick and of becoming deliberately sick in case of sick leave requests, unreasonable strictness by managers, continuous control of personnel regarding the use of equipment and personal protective equipment, intolerance of criticisms put forward by employees, and dealing with them in case of protest. One of the participants said the following about the fear of accountability by managers:

*“They did not divide the benefits and wages of Corona fairly. I was a complainer, and I even brought it up, but unfortunately, we are in a system where we follow up on financial issues a lot, and the managers will bring up a series of other issues. But in our administrative system, the officials have unwritten rules that no one can stick to them, and it’s interesting that if you have a trade union protest, they connect it to other issues and the subsequent troubles you say, do not worry about it.” (Participant 6)* [sic]

In addition to the heavy atmosphere created by the pandemic, the lack of provision of equipment and standard personal protective equipment by managers made working conditions more difficult for nurses. In this regard, one of the participants said the following:

*“At the beginning of the crisis, there was a shortage of personal protective equipment, and then there was no attempt by the managers to provide standardized personal protective equipment…masks were unfortunately not the standard of Covid-19 ward.” (Participant 7)* [sic]

Another issue that caused difficult working conditions for nurses was managers not paying heed to nurses’ demands and requests. One of the participants explained his experience as follows:

*“At the beginning of Corona, I said that I wanted to go to the emergency department from this department. I have been working here for seven years. The Matron said that you are running away. I said, Mr. Matron, I have been working for seven years, every written shift, every cooperation that had a problem, wherever there was a lack of workforce. Now I want to go to the emergency room, the CCU, and the ICU; I do not have any use here anymore.” (Participant 10)* [sic]

The participants claimed that nurses were ill-treated by managers, that nurses were accused of making themselves sick to use sick leave, and that there was a lack of support from managers. One of the participants explained it as follows:

*“If we got sick and applied for sick leave, the officials, especially the matron, would say that you are making yourself sick, go on sick leave. They no longer give sick leave to the result of PCR, and their trusted physician had to confirm that we were infected.” (Participant 10)* [sic]

#### 3.1.2 Team cooperation and communication challenges In difficult working conditions

During the COVID-19 outbreak, nurses either tried to help and cooperate with their colleagues or took mutual action against colleagues who were attempting to create tension and who refused to cooperate. Regarding the relative collaboration of nurses with each other, one of the participants said the following:

*“It has happened a lot that everyone does their work and then with the help of a busier colleague, and there is no additional pressure on us, it is perfect, and it feels good to work together, and we are not tired and have peace. I am happy that I fell in love with these colleagues and we work together. Sometimes some colleagues grumble, some cooperate well, good conditions are difficult, and people are different; their behavior is also different in difficulty.” (Participant 3)* [sic]

One of the problems of cooperation between nurses was unprofessional and tension-causing behavior between colleagues, which created an unpleasant work environment. In this regard, one of the participants said the following:

*“There is cooperation and non-cooperation. If they do not help me; I will not help them. This kind of unprofessional behavior in the work environment makes a person very nervous; For example, we draw lots to divide the work of patients, colleagues cheat so that they do not take sick patients, such small things make people nervous.” (Participant 8)* [sic]

#### 3.1.3 The shadow of burnout

For nurses, overcrowding of departments, high workload, and harsh working conditions caused a lack of motivation, excessive physical and mental fatigue caused by the job, the need to stay away from the work environment and rest, and the desire to leave the job. One of the participants said the following about excessive psychological and physical work fatigue:

*“Despite the hard-working conditions we had during the COVID-19 era, I was drained physically due to the high workload, and from a mental point of view, because young patients were dying or having problems at work and on the sidelines, it made me more tired.” (Participant 3)* [sic]

Another participant mentioned his desire to quit his job and said:

*“I’m tired of everything. I want to resign, but I’ve worked in this system for ten years. Hope for the future sometimes comes to me.” (Participant 10)*

One of the nurses explained the lack of motivation to work due to demanding working conditions as follows:

*“Despite the bad working conditions, the degradation of social status during the Covid-19 era, low salaries and benefits, and humiliation by the officials, I became completely unmotivated in my work.” (Participant 10)* [sic]

#### 3.1.4 Shortage inequity

During the COVID-19 outbreak, the working conditions of nurses became different than they had been previously. Critical conditions were associated with features such as a lack of workforce, a large number of nurses getting sick, a decrease in the quality of care, the occurrence of errors due to stress, constant contact with Covid patients, and a reduction in the quality of work of nurses due to stress. One of the participants, who was a Matron, explained the lack of workforce in this way:

*“The shortage of personnel was felt in COVID-19 couriers, and that was due to the illness of some colleagues and the lack of fresh personnel.” (Participant 9)* [sic]

In government hospitals, an unsafe work environment was created due to improper physical space, improper ventilation inside the wards, non-observance of environmental hygiene, inappropriate restrooms, lack of distance in the wards for patients, and lack of equipment and personal items. This made working conditions difficult. On the non-standard atmosphere of the departments and staff restrooms, one of the participants said the following:

*“We had the most infections in the restroom between shots, which was surprising in a row. All those who used the restroom tested positive within a week. Our next ward is the post-CCU ward, where mostly non-COVID-19 patients are hospitalized; our restroom in the ICU is shared with them, separated by two closets as long as one meter, which exchanges air from above. It would be best if you considered that our ward was Covid-19. There was a non-Covid ward where high-risk patients were hospitalized.” (Participant 6)* [sic]

A participant explained the spread of disease due to poor ventilation in the ward as follows:

*“Unfortunately, there was no proper ventilation in the department where we worked, and there was always a virus in the ward’s air, which created bad conditions for us and the patients.” (Participant 7)*

A participant described the harsh working conditions due to the lack of personal protective equipment and medical equipment early in the pandemic as follows:

*“The initial crisis was such that masks, protective clothing, and even gloves could not be found all of a sudden, and our hospital also faced a shortage, and we were forced to use quotas.” (Participant 7)* [sic]

#### 3.1.5 Dissatisfaction with unfair wages and benefits

Low salaries and benefits, differences in payments, and imbalance between working conditions and wages and benefits increased nurses’ worries in their work environment and even in their daily lives, and in some way, dissatisfaction was created among the nurses. One of the participants explained the imbalance between nurses’ working conditions and salaries and benefits as follows:

*“Unfortunately, our salaries increased a little, but not that much; for example, with the hard-working conditions we had and the deal was for our lives, we should have a much higher salary.” (Participant 8)* [sic]

One of the participants said the following about the low salaries and benefits, their reduction, and the resulting dissatisfaction:

*“The monthly salary that was our right! And it is too low, unfortunately. Managers said that salaries are paid on time. According to their own contradictory statements, they updated the system, but it was a third of a year ago. Well, the salaries and benefits had actually decreased, but the cost of living had increased. I mean, this situation of no money made me stressed.” (Participant 10)* [sic]

#### 3.1.6 Not having their work and sacrifice appreciated

Nurses understood and expressed the working conditions during the COVID-19 outbreak to be very similar to battlefield conditions so they saw themselves as being on the front line of the fight against coronavirus. According to the participants, they experienced this in terms of overcrowding in the wards, the number of patient deaths, and the deaths of colleagues. However, despite their dedication and sense of honor during this time, they claimed to feel that they were treated with indifference by the general population and officials. In comparison, soldiers were considered popular among the general population during wartime. In other words, the nurses experienced the feeling that the public and authorities did not understand their sacrifices, and this paradox intensified the insecurity and difficulty of working conditions for the nurses. One of the participants compared his and his colleagues’ working conditions and sacrifices to the war front as follows:

*“The situation was absolutely critical and we thought that we were in a war situation. I felt that now I am not speaking for myself, my colleagues were sacrificing more than they could.” (Participant 7)* [sic]

One of the participants explained his view on the popularity of soldiers compared to the rejection of nurses by the public and even their own families:

*“This situation is like wartime, with this difference, those who went to the front and returned, everyone went to meet them and make them happy. But we are very strange, we do not need to be taken over, at least they do not destroy and reject us.” (Participant 5)* [sic]

#### 3.1.7 Suffering and fatigue of using personal protective equipment (PPE)

Using personal protective equipment for a long time caused side effects such as fatigue, sweating, mask marks on the face, back pain, varicose veins, headache, and overall physical discomfort. One of the participants said the following about the discomfort of wearing non-standard clothes:

*“We had to use a protective cover, special warm clothes, some of which were non-standard and wearing them was painful. And also hats and masks for a long time that we did not take off these clothes even for a short time for fear of getting infected. In general, unfortunately, the coverage conditions were such that we were bothered.” (Participant 7)* [sic]

One of the participants explained the wound caused by the long-term use of a mask and the sensitivity caused by the use of low-quality and non-standard equipment as follows:

*“Exactly what the pictures and videos showed, the traces of the masks on the face and the wounds were created, it took half a day and a day to return to normal. Also, some gloves and clothes caused eczema and sensitivity on our skin.” (Participant 6)*

Another participant mentioned low back pain and varicose veins caused by hard work:

*“Physically, I said that we are worn out due to excessive fatigue and work pressure, I have back pain, I have varicose veins. This disease has psychological and physical effects as much as you want. Especially if your job is nursing and you have to be on your feet a lot, back pain and varicose veins are routine side effects.” (Participant 10)*

Another participant mentioned headaches caused by prolonged use of a mask:

*“And after the spread of covid-19, I get headaches that last for a certain number of hours or with a wave, because when we give old oxygen to the lungs, headaches occur, I have that hypoxia.” (Participant 6)* [sic]

### 3.2 The shadow of suffering and death

In addition to unsafe working conditions experienced by nurses during the Coronavirus pandemic, personal and family life became complex and challenging for nurses in the shadow of a lack of recreation and rest, fear of illness, imminent death, reduced resilience, and deteriorating living conditions.

#### 3.2.1 Deprivation of entertainment and rest

With the spread of COVID-19, the increase in the workload of nurses and the implementation of quarantine caused a reduction or cancelation of recreational programs such as sports programs, excursions, and travel. Therefore, this situation created a void of entertainment for nurses. One of the participants explained the lack of recreational, sports, and sightseeing programs as follows:

*“After the outbreak of covid-19, going to the market and shopping and such things, such places are almost canceled for us, unless we have to go out. The fun we had before, the park or a place to go for a walk, these are mostly canceled.” (Participant 1)* [sic]

A participant explained the lack of travel and recreation due to long shifts and the quarantine conditions of the disease as follows:

*“We nurses are currently unable to travel, both because of the spread of the disease and because of the many shifts. The working conditions have become so difficult due to the illness of colleagues and the lack of manpower, that we cannot think about fun and travel.” (Participant 4)*

#### 3.2.2 Fear of illness and imminent death

Fear of sickness and impending death is a mental state caused by the fear of contracting a disease and dying oneself a family member or friend contracting the disease, and fear of the death of loved ones. Along with psychological problems such as depression, loneliness, difficult living conditions, negative thoughts and obsessions, aggressiveness and lowering of tolerance thresholds, and generally, the regression of life affairs instead of progress, they cause a disturbance to life’s flow and everyday activities. One of the participants explained the fear of illness and imminent death due to infection in the following way:

*“I’ve been under a lot of mental stress since the severe lung complications caused by COVID-19; it always made me have nightmares that God forbid I get infected again, and this time I do not survive and die. In other words, I always feel doomed, and I think I will probably get stuck and die these days.” (Participant 8)* [sic]

A participant explained his fear of infecting his family and friends as follows:

*“I am afraid of my family, friends, and people who are dear to me getting sick. Now the fear is more because my job is related to health and care, but I cannot do anything in case of severe conflict with my loved ones. And it is not in my hands whether they get sick or recover, and God knows how stressful this situation is for me.” (Participant 15)* [sic]

Another participant said the following about the fear of premature death, either hisor his loved ones:

*“Once, my father got sick and his lungs were nearly 70% affected and he could not breathe well. My heart was empty and I said that he will die, I was very scared, I said that I wish I was in my father’s place. The stress was really deadly, I died and revived a thousand times in those few days.” (Participant 13)* [sic]

#### 3.2.3 Low resilience

Triggers such as psychological, social, and workload impositions on nurses during the COVID-19 pandemic caused mental fatigue, reduced their tolerance threshold, and led to the occurrence of reactions such as aggression and complaining about current conditions. One of the participants described the high workload and the resulting stress as follows:

*“I have the experience of heavy shifts. The patients were in a very bad condition, most of them were in a coma phase. Unfortunately, our shifts were back to back, well, I was always on shift, and I was dealing with a sick patient, and this caused a hidden stress to come to me.” (Participant 7)* [sic]

One of the participants mentioned the occurrence of aggression caused by the mental effects of dealing with COVID-19 as follows:

*“I was very sad when a young man suffered in front of my eyes, who was not like a cancer patient whose fate is likely to be known, this young man dies. A few days ago, two young patients died, I was very sad, I went home, I had become aggressive. These conditions affect our psyche, either we become aggressive or depressed.” (Participant 5)* [sic]

One of the participants explained the decrease in tolerance of the situation and the increase in his grumbling as follows:

*“I could not bear all these troubles in crowded couriers like the fifth courier. My sister’s husband was young, God’s servant died. At work, at home, instead of being a pillar for those around me, I was all complaining, why did this disease come. What sin did we commit?” (Participant 15)* [sic]

#### 3.2.4 Deteriorating conditions of life

The spread of the COVID-19 disease and the creation of difficult living conditions caused people to experience profound changes and challenges. These conditions included the fading of joys and the intrusion of hardships, leading to a transformative process that shaped how people move through their existence. Regarding the prevalence of fear and difficult life conditions, a participant noted the reduction of pleasurable activities and the poisoning of life as follows:

*“Well, all this stress and fear of getting infected disrupts life, I do not enjoy anything. I do not enjoy my work at work, I do not enjoy anything in my daily life, for me, life has become like hell.” (Participant 4)* [sic]

A participant explained the difficult living conditions after the outbreak of the disease as follows:

*“Living conditions had become extremely difficult, you experienced it yourself, and we could not even sit with our families without masks. The whole life plans for everyone and for me are messed up.” (Participant 11)* [sic]

## 4 Discussion

This study was conducted to discover nurses’ perceptions of work and life during the COVID-19 pandemic. This study showed that nurses experienced unsafe working conditions during the pandemic and simultaneously experienced and viewed personal and family life as hard-working conditions. Nurses were under all-round pressure and faced a bottleneck, as the number of patients increased sharply compared to before the pandemic. During the pandemic, there were tensions created in the work environment by managers and colleagues, hard and tiring working conditions, lack of workforce and equipment, unsafe work environment, low and unfair salaries and benefits, and a sense that their dedication was not appreciated. Many nurses considered themselves soldiers fighting against the coronavirus and expected constructive interaction and popularity from society and the managers of healthcare organizations, whereas early in the pandemic they experienced something different. In addition, the suffering, fatigue, and physical problems caused by the working conditions and the use of personal protective equipment caused nurses to feel insecure in the work environment. At the same time, the unavailability of recreation due to social restrictions and the high workload of nurses, the creation of distorted psychological conditions in the form of increased aggression, fear of contracting and dying from disease, and the decline of normal living conditions made life difficult for nurses. Requirements related to the work environment and daily life created tension and difficulty for nurses.

One of the results of this research was the belief held by nurses that they needed to be supported and understood by managers. Most of the nurses experienced the fear of being reprimanded by managers. Many studies pointed out the lack of support from managers and organizations, but no study specifically mentioned the fear of nurses being questioned by managers. In their research, Karimi et al. (20) noted the experience of nurses not being supported by managers. Of course, this result may have been obtained because it resulted from qualitative research in the field of work in Iranian hospitals. These conditions are caused by the heavy organizational atmosphere that managers sometimes apply in hospitals, resulting in job burnout and mental problems for nurses, in which case the provision of care services to patients will be disturbed and the quality will decrease (21). Most of the nurses had experienced a lack of standard personal protective equipment being provided by hospital managers. A study conducted in Saudi Arabia by Alruwaili and Baker (22) showed that nurses experienced dissatisfaction, insufficient support, and a strong sense of management deficiencies that could affect their presence at work. Moradi et al. (15) also pointed out that managers do not provide enough standard uniforms for nurses.

Another common experience among nurses was that managers ignored their demands. In their study, Digby et al. (23) mentioned that one of the factors involved in nurses’ well-being from the nurses’ point of view was when managers supported them by listening to and valuing their voices. Another common experience by most nurses has been the managers’ accusation that they are pretending to be sick to use sick leave. In their study, Guttormson et al. (24) examined the experiences of 498 special care nurses across the United States using open-ended questions in a virtual space. They mentioned the insufficient support of managers and organizations for nurses, which is in line with the results of our study (6). In the case of White et al. (7), in their qualitative study conducted with a phenomenological approach to discover the managers’ experiences during the COVID-19 pandemic, managers support nurses’ payment, respectful communication with nurses, and concern for improving conditions. Nurses’ work has been mentioned despite management challenges. These results, which are different from the results of the present study, are probably due to the type of communication culture between managers and nurses in their field of work (7).

The results of the present study showed a contradiction in how nurses interact with each other. In other words, nurses interact and cooperate in the work shift and workplace depending on the colleague’s behavior. That is, they cooperate with those colleagues who cooperate and show mutual behavior with them and not with those who create tension or do not cooperate with them. This conflict in the interaction of colleagues and the form of mutual action and reaction makes working conditions difficult for nurses. In their study, Yan et al. (25) pointed out the greater cooperation of healthcare workers, primarily nurses working in the care departments of patients with COVID-19, compared to other departments due to the workload’s unique working conditions and fatigue. The participants of this study also mentioned the cooperation between nurses. The results of a study conducted by Jerng et al. (26) showed that interpersonal conflicts among healthcare workers can negatively affect their well-being, and this is in line with the results of the present study, which showed that nurses experience non-cooperation and tension created by some other nurses. To improve collaboration among nurses and reduce interpersonal conflicts, healthcare organizations should create a supportive work environment, provide clear rules and procedures to prevent negative behavior, and promote mutual respect among healthcare workers (26, 27).

Nurses have been on the front lines of the fight against COVID-19 and care for hospital patients for long hours (28). The pandemic increased nurses’ workload and stress levels, leading to physical and mental fatigue (29). In the present study, it was found that the working conditions in the field of nursing, due to the crowded departments, high workload, and challenging situations, caused a lack of motivation and excessive physical and mental fatigue in nurses, and finally led to the creation of an exhausting and unbearable work environment. A study conducted during the COVID-19 pandemic by Conolly et al. (30) found that the increased stress and workload among nurses hurt their motivation and performance and caused a double reason to leave their jobs. The results of Nagel et al.’s study (31) also confirmed these findings and stated that during the COVID-19 pandemic, nurses faced a lot of workload and stress due to low human resources.

Results of other studies emphasize the importance of adequate personal protective equipment (PPE), rest areas, and ventilation in healthcare settings to prevent the spread of airborne diseases (32, 33). While there are general guidelines, the unprecedented pandemic overwhelmed existing infrastructure. Findings from studies show that unprepared healthcare systems and shortages of PPE/facilities increase the risk of infection among frontline workers (34). This is consistent with the results of the present study, which shows that the lack of personal equipment, improper ventilation, and non-standard space of wards and personnel rest rooms cause a double burden on the mental pressure that nurses suffer. In this regard, the study of Lesan et al. (35) confirms these findings because the small and non-standard rest spaces and the lack of proper ventilation cause tension among health workers.

The COVID-19 pandemic has significantly affected the working conditions of nurses and has led to an increase in workload and stress. This has led to an imbalance between working conditions and salaries and benefits. Low salaries, benefits, and mental and physical problems caused by the epidemic have led to nurses’ dissatisfaction (36). This study showed that nurses face low salaries and benefits, unfair payments, and an imbalance between working conditions and salaries. This is contrary to the study conducted by Morshedi et al. (37) in Iran, which showed that most nurses were satisfied with the timely payment of their salaries and benefits. However, they still complained about the discrepancy between their salary and workload, and this situation may be different depending on the location and specific health institutions before and after the COVID-19 pandemic (37). In line with the results of the present study, the study of Şahin et al. (38) conducted in Turkey confirmed that nurses faced insufficient and unfair payment of salaries and benefits during the COVID-19 pandemic. Therefore, to meet these challenges, it is necessary to improve policies and mobilize existing resources and measures, such as increasing salaries, ensuring timely provision of benefits, and more support for nurses, as these measures can help improve the working conditions of nurses (39). Contrary to the results of the present study, a survey conducted by Şahin et al. (2020) found that nurses and doctors are supported, respected, and described as heroes by Turkish society and that they are appreciated by the general population. This is contrary to the findings of the present study. This divergence can be due to cultural differences and the different policies of two different cultures.

The results of the present study showed that nurses compare their work to being on the “battlefield” due to high workload, emotional stress, and risks to personal safety. Therefore, the participants acknowledged that their sacrifices remained unrecognized and were not appreciated as they deserved. Galehdar et al. (40) found that nurses often face challenges of underappreciation and appreciation, which can negatively affect their motivation and job satisfaction, consistent with this study’s results. The results of the study conducted by Kalateh Sadati et al. (10) also emphasized the increased support for nurses during the pandemic and suggested that improved public awareness is needed to encourage and appreciate healthcare workers. In addition, the findings of Hou et al. (41) suggested that more attention be paid to recognizing and appreciating the sacrifices of nurses through gestures of gratitude and support as this can help solve this specific concern of nurses.

The present study identified suffering and fatigue caused by personal protective equipment as essential challenges for nurses during the COVID-19 pandemic. A survey by Aloweni et al. (42) found that long-term use of PPE resulted in adverse effects such as pressure injuries, mask-induced acne, and irritation or pain, especially when using N95 masks. These side effects have negatively affected the work of healthcare workers, with women and those working in high-risk sectors being more exposed to PPE-related side effects (42). Jose et al. (43) also confirmed these findings and stated that long-term use of personal protective equipment can lead to physical discomfort, skin problems, and increased fatigue. To address these challenges, healthcare systems should implement clear and practical training and education programs, ensure adequate access to comfortable and safe protective equipment, promote a positive environment to improve adherence to safety protocols and the appropriate use of personal protective equipment, and provide regular breaks in the shift. Some change is necessary to improve the working conditions of healthcare workers (44).

The results of the present study have shown that nurses have experienced a lack of rest and recreational time in their lives. In this regard, the results of other studies have shown that the pandemic led to risky working conditions, unconventional work schedules, reduced rest time, and increased working hours for nurses. In addition, with the increased workload and quarantine measures, this pandemic caused nurses to reduce or cancel their previous leisure activities, such as sports, tourism, and social outings. It changed the quality of life of nurses (45, 46). In this regard, promoting work-life balance, supporting self-care activities, and fostering a culture of well-being is very important to address this issue (47–49).

The results of the present study showed that fear of illness and imminent death was a common source of stress for nurses during the COVID-19 pandemic. In this regard, the study by Jafar et al. (50) showed that healthcare workers, including nurses, face challenges in their performance due to the fear of contracting COVID-19 and its adverse effects on their work. This supports the present study’s findings, as fear of illness and death may negatively affect nurses’ performance during a pandemic. The study of Mayer et al. (51) also confirms the present study’s findings because the fear of infecting family members has been an essential concern for healthcare workers, including nurses, during the COVID-19 pandemic. Therefore, the managers of medical centers must pay attention to this issue and apply efficient planning to protect nurses from the stress caused by the fear of contracting COVID-19.

Low resilience was a shared experience among nurses in the present study. The prolonged and severe nature of the pandemic caused many challenges to nurses’ flexibility and coping mechanisms (52). The results of the study conducted by Shah et al. (53) during the COVID-19 pandemic aimed to determine the effect of psychological fatigue on the relationship between work stress, anger, and absenteeism among nursing staff. The results showed that factors such as job stress and anger contribute to emotional exhaustion, and in addition, irritation may cause adverse interactions among employees and encourage staff to express aggressive behaviors (53, 54). In their study, Forrest et al. (55) reported that in the early phase of the pandemic, 15% of 14,600 healthcare workers experienced anger the day before completing the survey. Hence, occupational protocols and administrative strategies are needed to target safety issues, monitoring, incident referral, and workforce training on preventing and managing workplace anger and aggression (56). However, the results of these quantitative studies have yet to provide a correct understanding of the lived experiences of nurses. Hence, nurses’ experience of low resilience includes aggression due to demanding working conditions and fatigue, and their tolerance threshold has decreased.

Finally, the findings showed that nurses undergo a transformation process amid adversity because the outbreak of the COVID-19 disease created difficult living conditions with a decrease in pleasurable moments and an increase in problems; in other words, they have experienced declining life conditions. Although these results have not been explicitly mentioned in any study during the pandemic, nurses faced many challenges, such as fear of infection, stress, anxiety, and depression, that disrupted their daily lives and affected their mental health (57). Rasmussen et al.’s study (58) showed that nurses during the pandemic had to deal with various loads, including professional relationship load, personal load, environmental load, physical symptom load, and emotional load. Mohammadi et al.’s study (59) showed that the pandemic affected nurses’ quality of life.

Overall, the in-depth review of this study showed that nurses had very high difficulty working and living conditions in Iran during the COVID-19 pandemic. Insecurity and stress prevailed both in their work environment and personal lives. The limitations of this study include the lack of quick access to the participants due to the high workload and the lack of full observation of the body language of the participants in virtual interviews. The findings of this study can be utilized to improve the approach of nursing managers, and nurses themselves to recognize the barriers they encounter and how they can take action to deal with them appropriately. Moreover, these findings can provide valuable insights for the development of additional qualitative and quantitative studies focused on examining the diverse aspects of the pandemic crisis risk and implementing measures to alleviate its detrimental impact on nurses. Healthcare organizations should urgently address the factors undermining nurses’ occupational safety and well-being by improving management support, facilitating teamwork, reducing burnout, providing financial aid, and providing psychological services. Recognizing the sacrifices of nurses through social gratitude is also recommended. Global healthcare systems must also focus on maintaining and caring for the nursing workforce as fundamental to successful pandemic responses.

## 5 Conclusion

The findings of this study refer to the perceptions and experiences of nurses regarding working and living conditions during the COVID-19 pandemic. Moreover, the findings refer to the challenges of the work environment for nurses caused by inefficient management, conflicting colleagues, challenging work environments, changing living conditions, and the manifestation of stress in the lives of nurses. The COVID-19 pandemic created many problems for nurses. To improve the working conditions of nurses and maintain their health, healthcare managers, government officials, and society must provide essential support in various fields, including providing sufficient resources and equipment, improving their work environment, and promoting mental health and proper appreciation of the efforts of nurses.

## Data availability statement

The original contributions presented in the study are included in the article/supplementary material, further inquiries can be directed to the corresponding author.

## Ethics statement

This study has been approved by the Ethics Committee of Tarbiat Modares University with the ethics code IR.MODARES.REC.1399.157. The method and instructions for conducting this study are by the provisions of the 1995 Helsinki Declaration, edited in 2001. In addition, written informed consent was obtained from all the participants. Participants were assured that their names would remain confidential.

## Author contributions

EsM: Conceptualization, Data curation, Formal analysis, Investigation, Methodology, Resources, Validation, Visualization, Writing – original draft, Writing – review & editing. ZV: Conceptualization, Formal analysis, Funding acquisition, Methodology, Project administration, Supervision, Validation, Visualization, Writing – review & editing. EeM: Conceptualization, Formal analysis, Investigation, Methodology, Supervision, Validation, Visualization, Writing – review & editing.
